# Supplementary material for: Kidney protection strategy lowers the risk of contrast-associated acute kidney injury
Source: PLoS One. 2024 Oct 24;19(10):e0312618. doi: 10.1371/journal.pone.0312618 (PMC11500849; doi:10.1371/journal.pone.0312618)
Supplement: S1 Table — (DOCX) [file pone.0312618.s001.docx]

**S1 Table.** Associated factors of contrast-associated acute kidney injury

| Variable | Odds ratio (95% CI) | *P* value |
| --- | --- | --- |
| Univariate analysis |  |  |
| KPS | 0.32 (0.05–2.02) | 0.225 |
| Isotonic saline hydration | 0.41 (0.11–1.51) | 0.182 |
| Contrast volume, per 10 mL | 1.09 (0.97–1.23) | 0.139 |
| CG ratio ≤1 | 0.47 (0.13–1.68) | 0.248 |
| Acetylcysteine | 0.56 (0.14–2.19) | 0.402 |
| Femoral access | 5.76 (1.44–22.99) | 0.013 |
| Emergency procedure | 1.57 (0.38–6.53) | 0.539 |
| Ad hoc PCI | 1.18 (0.16–8.79) | 0.875 |
| High or very high Mehran 2 risk category | 2.68 (0.69–10.49) | 0.156 |
| Clinical presentation (Reference: US/CCS) | | |
| Heart failure | 1.07 (0.30–3.78) | 0.917 |
| Acute myocardial infarction | 1.31 (0.37–4.61) | 0.678 |
| EGFR < 30 ml/min/1.73 m^2^ at Cath | 2.11 (0.58–7.68) | 0.259 |
| Diabetes mellitus | 0.69 (0.18–2.57) | 0.576 |
| Age ≥75 years | 0.94 (0.27–3.30) | 0.917 |
| LVEF <40% | 2.65 (0.74–9.54) | 0.136 |
| Multivessel disease | 10.25 (0.53–197.91) | 0.123 |
| Multivariate analysis* |  |  |
| Femoral access | 5.29 (1.24–22.49) | 0.024 |
| LVEF <40% | 2.94 (0.72–12.08) | 0.135 |
| Multivessel disease | 6.79 (0.34–134.41) | 0.209 |

Abbreviations: CI, confidence interval; KPS, kidney protection strategy; UC, usual care; CG ratio, contrast/estimated glomerular filtration rate ratio; PCI, percutaneous coronary intervention; US, unstable angina; CCS, chronic coronary syndrome; LVEF, left ventricular ejection fraction.

* Variables with *P* value <0.15 are included in the multivariable model.
