# Supplementary material for: Kidney protection strategy lowers the risk of contrast-associated acute kidney injury
Source: PLoS One. 2024 Oct 24;19(10):e0312618. doi: 10.1371/journal.pone.0312618 (PMC11500849; doi:10.1371/journal.pone.0312618)
Supplement: S1 Fig — (DOCX) [file pone.0312618.s002.docx]

**S1 Fig.** Workflow of the study protocol


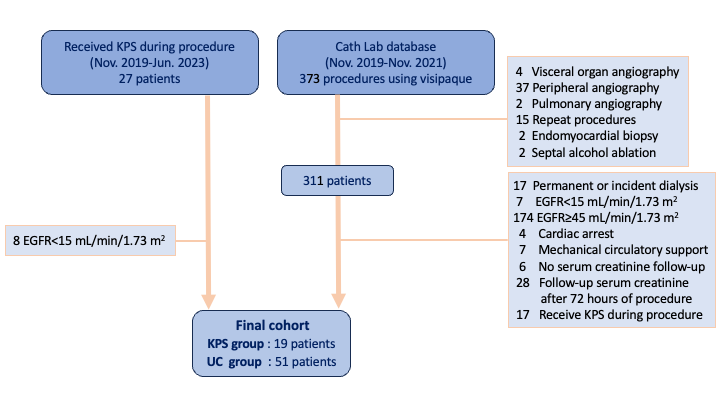


This study enrolled patients from two databases: those registered in the catheterization laboratory database and those who underwent KPS during CAG performed by our study team. In the catheterization laboratory database, 313 patients who received Visipaque 320 administration during CAG with a diagnosis of acute coronary syndrome, chronic coronary syndrome, heart failure, or moderate-severe valvular heart disease were initially found. Patients with eGFR <15 or ≥45 ml/min/1.73 m^2^, those who underwent dialysis before CAG, suffered from cardiac arrest, received mechanical circulatory support (including extracorporeal membrane oxygenation and intraaortic balloon pump) before or during CAG, and had unavailable follow-up serum creatinine values were excluded. Seventeen patients in the catheterization laboratory database were reclassified into the KPS group because they had undergone KPS during CAG. Notably, all patients in the KPS group had a follow-up serum creatinine value; eight were excluded because of an eGFR <15 ml/min/1.73 m^2^. Finally, 19 and 51 patients in the KPS and usual care groups, respectively, were included in this study.

CAG, coronary angiography; eGFR, estimated glomerular filtrate rate; KPS, kidney protection strategy; UC, usual care
