# Supplementary material for: Kidney protection strategy lowers the risk of contrast-associated acute kidney injury
Source: PLoS One. 2024 Oct 24;19(10):e0312618. doi: 10.1371/journal.pone.0312618 (PMC11500849; doi:10.1371/journal.pone.0312618)
Supplement: S2 Fig — Predicted and actual CA-AKI risks in different Mehran 2 risk categories in the entire cohort (a), KPS group (b) and UC group (c). (DOCX) [file pone.0312618.s003.docx]

**S2 Fig.** Predicted and actual CA-AKI risks in different Mehran 2 risk categories in the entire cohort (a), KPS group (b) and UC group (c).


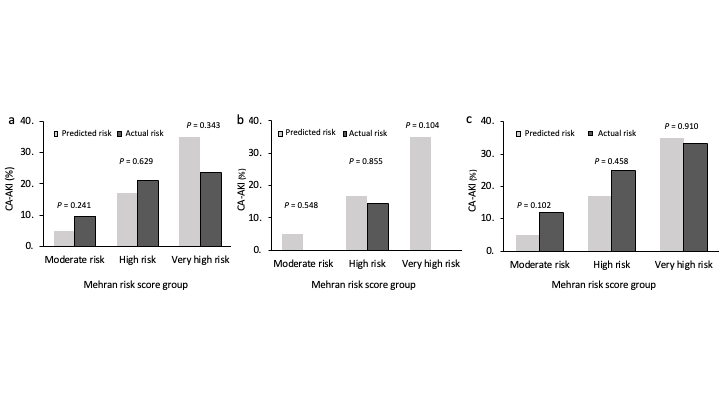


CA-AKI risk escalates across different risk categories, and the risk is consistent with Mehran’s study group’s initial report. Two patients with mild risk in the UC group were not included in the analysis.

CA-AKI, contrast-associated acute kidney injury; KPS, kidney protection strategy; UC, usual care.
