# Supplementary material for: Kidney protection strategy lowers the risk of contrast-associated acute kidney injury
Source: PLoS One. 2024 Oct 24;19(10):e0312618. doi: 10.1371/journal.pone.0312618 (PMC11500849; doi:10.1371/journal.pone.0312618)
Supplement: Attachment — Submitted filename: Response_to_Reviewers.docx [file pone.0312618.s004.docx]

Response to Reviewers

Reviewer #1: Dear editor,
first of all thank you for asking me to review this manuscript about kidney protection strategy to lower contrast induced kidney injury. To date, this is a extremely hot topic.
For the regards the content:
1. Can the authors be more specific about patients enrolled? Are all ST elevation myocardial infarction patients? or not? The population involved in this study is not clear: did the authors compare STEMI patients with patients that underwent staged procedures?

Reply：

Thank you for this suggestion. In this study, we enrolled patients diagnosed with acute coronary syndrome, chronic coronary syndrome, and heart failure. Of the total participants, 9 patients presented with ST-elevation myocardial infarction (STEMI), and 7 patients underwent primary percutaneous coronary intervention (PCI), with 1 patient in the KPS group and 6 patients in the UC group. Additionally, one patient with STEMI underwent staged PCI in the KPS group, while another patient in the UC group underwent staged coronary artery bypass grafting (CABG).

For patients with STEMI, the reason for delayed revascularization was good distal perfusion of the culprit vessel, which was perfused by collateral circulation. It is important to note that the number of patients with STEMI was relatively small, with only 2 patients in the KPS group and 7 patients in the UC group, making it challenging to compare outcomes with those of patients who underwent staged procedures. We have revised the inclusion criteria in the Methods section, and a comparison of STEMI presentations is presented in the revised Table 1.

[Page 6, Line 82] The inclusion criteria were Visipaque 320 administration, eGFR of <45 mL/min/1.73 m^2^ during CAG, and CAG indication with acute coronary syndrome, chronic coronary syndrome, and heart failure.

Revised Table 1 Baseline demographics and clinical characteristics of the patients

| Variable | KPS  (*n* = 19) | UC  (*n* = 51) | *P* value |
| --- | --- | --- | --- |
| Clinical presentation |  |  | 0.900 |
| STEMI | 2 (10.5) | 7 (13.7) |  |
| NSTEMI | 6 (31.6) | 19 (37.3) |  |
| Heart failure | 10 (52.6) | 21 (41.2) |  |
| US/CCS | 1 (5.3) | 4 (7.8) |  |

Why did the authors report "data not shown"?

Reply：

Thanks for your query. The “data not shown” mean the relevant data were not shown in the presented Table or Figure. To avoid misunderstandings, these sentences have been rephrased as:

[Page 16, line 190] Overall, the median Mehran 2 risk score was 8 (interquartile range [IQR]: 6–11.25), and approximately 51% of the patients were classified into high- and very high-risk categories.

[Page 20, line 265] Currently, all patients in the KPS group received ULCAG, and one-third of patients underwent staging procedures, such as zero contrast PCI and cardiac surgery, a few days later.

The number of patients is not enough to consider the strategy mentioned safety.

Reply：

Thank you for your comments. We agree that the safety of this strategy is uncertain. The sentence has been revised as follows.

[Page 19, line 247] This modified protocol was feasible for emergency procedures and in patients with medically controlled severe valvular heart disease.

In literature there are other trials comparing LVEDP vs UFR guided hydration with a lot of patients or comparing the type of contrast used, what is your novelty?

Reply：

Thanks for your query. Several hydration strategies have been proposed, and their efficacy in preventing CA-AKI has been demonstrated in recent clinical trials. However, the contrast volume was higher in these clinical trials than that in our study. (Please refer to the table below)

| Trials | Age | Estimated GFR | Contrast volume | History of heart failure |
| --- | --- | --- | --- | --- |
| POSEIDON | 71±9 | 48±9 | 104(84-187) | 31 (16%) |
| Geng Qian et al | 64±10 | 36 (23-48) | 161±67 | 132 (100%) |
| HYDRA | 68±10 | 73±27 | 135±94 | 36 (5.1%) |
| AVERT | 71±9.3 | 45.6±10.7 | 85.6±50.5 | 89 (31.1) |
| REMEDIAL III | 74±8 | 36±3 | 72±49 | NA |
| STRENTGH | 79.1±8.8 | 32±9 | 116±68 | 43 (35.2%) |
| NTUH-HC (KPS group) | 73.4±10.6 | 29.1±8.1 | 7.5(5-11) | 16 (84.2%) |

An ultralow contrast strategy was recommended in the KPS group in our study (Table 2). Both estimated GFR and contrast volume were the lowest in our study, in comparison with other clinical trials. (Please refer to table above). To our knowledge, this is the first study to show the cumulative effect of LVEDP-guided hydration and ULCAG in reducing CA-AKI in patients with concomitant high-risk conditions and KI.

Minor issues: need to check punctuation.

Reply：

Thank you for this suggestion. The punctuation was checked and corrected.

Reviewer #2: In this small sample retrospective study, the authors investigated the effectiveness of a comprehensive kidney protection (KPS) strategy compared to usual care (UC) in reducing contrast-associated acute kidney injury (CA-AKI) in patients with an eGFR rate between 15-45 mL/min who received coronary angiography (CAG). The KPS approach resulted in lower contrast media exposure and a higher proportion of patients receiving a hydration schedule with saline administration guided by left ventricular end-diastolic pressure (LVEDP). Although there is conflicting data on using N-acetylcysteine (NAC) in preventing CA-AKI, some studies suggest that administering NAC with crystalloids before kidney insult onset could prevent CA-AKI. In this study, a significantly higher proportion (89.5%) of KPS patients received NAC than UC patients (21.6%). The authors should explain why the higher NAC administration in KPS patients did not correlate with a lower incidence of CA-AKI.

Reply：

Thank you for your comments. We would like to clarify a few points regarding our study of the association between NAC administration (acetylcysteine) and the risk of CA-AKI.

First, we conducted an analysis using the entire cohort, including both the KPS and UC groups, to assess this association. The results suggested a protective effect of NAC administration with an odds ratio of 0.56, although this finding was not statistically significant. We attribute this lack of significance to the limited sample size in our study (S1 Table).

Second, in our primary analysis, which was performed on the IPTW-adjusted cohort, we found a significant reduction in the risk of CA-AKI in patients receiving KPS compared with those in the UC group. The odds ratio was 0.19 with a p-value of 0.009 (Figure 2). We believe that this difference was likely due to the higher frequency of NAC administration in the KPS group.

We appreciate your input and plan to address the limitations of our study, such as its small sample size.

Reviewer #3: In the presented study, Jong CB and co-authors investigated the effect of a novel comprehensive kidney protection strategy (KPS) on the risk of developing contrast-associated acute kidney injury (CA-AKI). This was a single-center historical cohort study of 70 patients with moderate to advanced chronic kidney disease (eGFR 15-45 ml/min/1.73 m2) who had undergone invasive cardiac catheterization – coronary angiography (CAG): 19 patients received the KPS strategy and 51 received standard care. In the adjusted analysis, the KPS group had a significantly lower risk of CA-AKI than the standard treatment group (4% vs. 20.4%; odds ratio 0.19). This effect was consistent across different patients subgroups, including elderly patients, acute myocardial infarction, advanced CKD, high-risk category, left ventricular systolic dysfunction, and multivessel disease.
The manuscript is generally well written. Since the effect of iodinated contrast agents on the development of CA-AKI is still controversial, this study is of potential interest. However, I would have further comments and suggestions as the presented study has several limitations and problems that should be addressed.

Main Comments

1. Concept of contrast-associated acute kidney injury and prevention strategies
The concept and scientific evidence for CA-AKI has changed in recent years, as the causal association of contrast agents with AKI is not clear. Accordingly, the consensus-based recommendations for their prevention and treatment have evolved. This should be briefly outlined in the introduction section of the manuscript.

Reply：

Thank you for your constructive suggestion. The Introduction section has been revised as follows:

[Page 4, line 56] The causal association of contrast agents with AKI remained controversial in recent years. The role of contrast agents in the development of AKI may be overestimated, especially in cases of multi-comorbidity risks. Therefore, this study stratified the risk of AKI according to a novel predictive model that included eight clinically important variables, with the exception of the contrast volume. Herein, a comprehensive kidney protection strategy (KPS) that combines the concepts of LVEDP-guided isotonic saline hydration, ULCAG, and a staged coronary revascularization procedure under suitable conditions was introduced.

2. Study population
a. It is not clear from the data presented how patients were selected for the new KPS strategy compared to standard-of-care strategy for the prevention of CA-AKI. Based on which baseline characteristics? This could lead to selection bias, which is a significant limitation of the presented study.

Reply：

Thank you for your comments. All patients referred to our study team for coronary angiography (CAG) underwent kidney protective strategies (KPS) if they had comorbidities and an estimated glomerular filtration rate (eGFR) of less than 45 mL/min/1.73 m^2^. Patients with kidney insufficiency (KI) who were referred to other interventionists and underwent similar procedures but did not receive KPS during CAG were categorized as the usual care (UC) group. The unintentional referral of patients may have contributed to the similar baseline characteristics between the two groups, except for the diagnosis of multivessel coronary artery disease, which was determined after invasive CAG. However, this may have introduced selection bias, and therefore, the Methods and Limitations sections have been revised to address this concern.

[Page 5, Line 68] All patients referred to our study team for CAG underwent KPS, while comorbidity with an eGFR of <45 mL/min/1.73 m^2^. In contrast, patients with KI who were referred to other interventionists and underwent similar procedures but did not receive KPS during CAG were categorized into the usual care (UC) group. This study enrolled patients from two databases: those registered in the catheterization laboratory database and those who underwent KPS during CAG performed by the study team. The enrollment periods of the two databases overlapped, and 17 patients in the catheterization laboratory database were reclassified into the KPS group because they had undergone KPS during CAG. (S1 Fig.)

[Page 20, Line 272] This study has few limitations. First, the influence of residual confounding factors and selection and indication biases are unavoidable in observational studies.

b. The authors have presented a patient inclusion flowchart in Supplementary Figure 1. It appears that 17 patients were excluded from the standard treatment group because they received a KPS strategy during the CAG procedure. Why were these patients included in the standard treatment group in the first place and not in the KPS strategy group? This should be clarified.

Reply：

Thank you for this suggestion. The Methods section has been revised as follows:

[Page 5, Line 68] All patients referred to our study team for CAG would undergo KPS, while comorbidity with an eGFR of <45 mL/min/1.73 m^2^. In contrast, patients with KI who were referred to other interventionists and underwent similar procedures but did not receive KPS during CAG were categorized into the usual care (UC) group. This study enrolled patients from two databases: those registered in the catheterization laboratory database and those who underwent KPS during CAG performed by the study team. The enrollment periods of the two databases overlapped, and 17 patients in the catheterization laboratory database were reclassified into the KPS group because they had undergone KPS during CAG. (S1 Fig.)

3. Standard-of-care strategy
The authors should present the standard- of-care strategy for the prevention of CA-AKI in more detail. How were patients selected for standard treatment?

Reply：

Thank you for this suggestion. Patients with KI who were referred to an interventionist other than our study team but did not receive KPS during CAG were categorized into the usual care (UC) group. The Methods section has been revised, and the UC strategy has been introduced in more detail. Notably, the strategy for CA-AKI prevention in the UC group was not standardized in all patients, which reflects the real-world situation in the daily catheterization room. For example, the rate of hydration was significantly lower in the UC group, wherein two-thirds of patients had a history of HF. Most non-hydrated patients may be concerned about fluid overload, which reflects the real-world situation in clinical practice. Therefore, we used the term UC instead of the standard-of-care in our study.

[Page 8, Line 115]

***UC strategy***

In contrast, patients receiving UC were hydrated with an isotonic saline infusion at a constant rate of 0.5 or 1 mL/kg/h in those with or without heart failure (HF) before and after 12 h of contrast exposure [8]. Notably, the strategy for CA-AKI prevention in the UC group was not standardized in all patients, which reflects the real-world situation in the daily catheterization room. Some patients may not have received saline hydration because of concerns regarding fluid overload. Minimum contrast volume administration and decision on the timing of coronary revascularization were not standardized; they depended on the operator’s discretion.

4. Pre-existing kidney disease vs. acute kidney injury
The authors should explain in more detail whether the patients with an eGFR of <45 mL/min/1.73 m2 were patients with moderate to advanced CKD (stages G3b and G4) or whether patients with AKI and an eGFR of <45 mL/min/1.73 m2 were also included. This should be clarified.

Reply：

Thank you for your insightful comment. The inclusion criteria of our study were Visipaque 320 administration and eGFR <45 mL/min/1.73 m^2^ during CAG. We have rephrased the term of CKD to ‘eGFR at cath’ as half of the patient have not data of baseline eGFR (determined by prior 3-6 months of CAG). The baseline eGFR data are provided in revised Table 1. Owing to the lack of baseline eGFR data, the proportion of patients with AKI or CKD was uncountable. However, we have presented the follow-up kidney function status (i.e., improved, stable, and deteriorated) in the revised Table 2, and nearly one-third of the patients had improved kidney function in both groups without significant differences. Based on this result, we assumed that the proportion of AKI in both groups might be similar and have less impact on the risk of CA-AKI. Consistently, the kidney function reported in Mehran’s study was the serum creatinine measured at admission or on the morning of the PCI procedure; the creatinine level prior to 3 months of the procedure was not reported. Therefore, the kidney function in the current study was consistent with the Mehran 2 risk scoring system. However, this important issue has been mentioned in the Limitations section. Furthermore, there was only one patient in the KPS group with a baseline eGFR <30 ml/min/1.73 m², rendering the subgroup analysis inapplicable.

Revised Table 1 Baseline demographics and clinical characteristics of the patients

| Variable | KPS  (*n* = 19) | UC  (*n* = 51) | *P* value |
| --- | --- | --- | --- |
| Serum creatine at cath, mg/dL | 2.4 ± 0.7 | 2.3 ± 0.7 | 0.696 |
| Mean eGFR at cath, ml/min/1.73 m^2^ | 29.1 ± 8.1 | 30.0 ± 8.7 | 0.707 |
| eGFR 30-45 at cath, mL/min/1.73 m^2^ | 10 (52.6) | 27 (52.9) | 1.000 |
| eGFR <30 at cath, mL/min/1.73 m^2^ | 9 (47.4) | 24 (47.1) |  |
| Baseline eGFR, mL/min/1.73 m^2^ (*n* = 35) | 36.9 ± 11.7 (*n* = 5) | 36.9 ± 11.7 (*n* = 30) | 0.680 |

Revised Table 2 Characteristics of KPS and CA-AKI in the patients

| Variable | KPS  (*n* = 19) | UC  (*n* = 51) | *P* value |
| --- | --- | --- | --- |
| Kidney function after day 1-3 of contrast exposure |  |  | 0.380 |
| Improved | 6 (31.6) | 16 (31.4) |  |
| Stable | 12 (63.2) | 25 (49.0) |  |
| AKI (deteriorated) | 1 (5.3) | 10 (19.6) |  |

[Page 21, Line 277] Third, half of the patients did not have baseline eGFR data, which implies that some of the patients may have developed AKI during CAG. However, the follow-up kidney function after CAG showed that nearly one-third of the patients had improved kidney function in both groups without a significant difference. Based on this result, we assumed that the proportion of AKI in both groups might be similar and have less impact on the risk of CA-AKI.

5. KPS protocol and contrast volume
According to the KPS protection protocol, a maximum contrast volume equal to or less than the last numerical eGFR value was used to minimize contrast administration during CAG. Did all patients undergo diagnostic CAG only or were there patients (in the KPS group and in the standard treatment group) who underwent coronary intervention with balloon angioplasty and stenting immediately following the diagnostic procedure? In this case, the contrast volume is much higher. This should be explained in more detail.

Reply：

Thank you for this suggestion. The proportion of ad hoc PCI (mean immediate PCI after CAG without staging) is shown in Table1. There was a numerically higher proportion of ad hoc PCI in the UC group, but the difference was not statistically significant. Although there was a significantly higher contrast volume administration in the UC group, the univariate analysis of the entire cohort showed that neither contrast volume nor ad hoc PCI was associated with the incidence of CA-AKI. (Revised S1 Table)

**Table 1**. Baseline demographics and clinical characteristics of the patients

| Variable | KPS  (*n* = 19) | UC  (*n* = 51) | *P* value |
| --- | --- | --- | --- |
| Cardiac catheterization |  |  |  |
| Ad hoc PCI | 2 (10.5) | 15 (29.4) | 0.127 |

**Revised S1 Table.** Associated factors of contrast-associated acute kidney injury

| Variable | Odds ratio (95% CI) | *P* value |
| --- | --- | --- |
| Univariate analysis |  |  |
| Contrast volume, per 10 mL | 1.09 (0.97–1.23) | 0.139 |
| CG ratio ≤1 | 0.47 (0.13–1.68) | 0.248 |
| Ad hoc PCI | 1.18 (0.16–8.79) | 0.875 |

6. CA-AKI
As already mentioned, the causal attribution of contrast agents leading to AKI is not clear. Based on the follow-up of serum creatinine on days 1 and 3 after contrast exposure, the authors should also report and compare the proportions of patients with stable kidney function and a decrease in serum creatinine concentration of at least 0.3 mg/dL in both groups (and not just an increase in serum creatinine) during the first 3 days after contrast exposure (these data should be presented in Table 2).

Reply：

Thank you for this suggestion. We have provided the follow-up kidney function status (i.e., improved, stable, and deteriorated) in revised Table 2.

Revised Table 2 Characteristics of KPS and CA-AKI in the patients

| Variable | KPS  (*n* = 19) | UC  (*n* = 51) | *P* value |
| --- | --- | --- | --- |
| Kidney function after day 1-3 of contrast exposure |  |  | 0.380 |
| Improved | 6 (31.6) | 16 (31.4) |  |
| Stable | 12 (63.2) | 25 (49.0) |  |
| AKI (deteriorated) | 1 (5.3) | 10 (19.6) |  |

7. Baseline patient characteristics and risk for CA-AKI
Based on the data presented in Table 1, a significantly higher proportion of patients in the standard-of-care group had multivessel disease and more complex coronary artery disease anatomy. Nearly 30% of patients in the standard group underwent immediate PCI compared to 10% of patients in the KPS group. In addition, patients in the standard group were less likely to receive isotonic saline protection, which is the standard-of-care for prevention of CA-AKI. All in all, it is not surprising that patients in the standard treatment group received a higher volume of contrast medium, had a higher baseline risk and thus a higher incidence of CA-AKI (independent of statistical adjustments). This is confirmed by the unadjusted baseline risk of CA-AKI, where patients in the standard treatment group had a numerically higher baseline risk of CA-AKI. Due to the small number of patients in both groups, all calculations of statistical differences and statistical adjustments could be associated with a type 2 statistical error.

Reply：

We appreciate your insightful comments. It is true that patients in the UC group received a higher volume of contrast medium and had a substantially higher proportion of multi-vessel disease and ad hoc PCI, as shown in Tables 1 and 2. These procedural variables were included in Model 2 of the Mehran 2 risk score. According to the large cohort in Mehran’s report, Model 2 had a slightly higher discriminative power than Model 1. (C-statistic: 0.72 -> 0.74 in the derivation cohort, 0.84 ->0.86 in the validation cohort), and the role of contrast volume<100 mL and complex coronary artery anatomy had only a few impacts on the risk of CA-AKI compared with other variables in model 2, as shown in table below.

Therefore, we propose that the risk of CA-AKI should be evaluated according to the Mehran 2 risk-scoring system, not an individual variable. The initial Mehran risk category and risk score (pre-IPTW) were higher in the KPS group than in the UC group (left panels in Fig 1 A and B). After adjusting for the Mehran risk category with IPTW, the Mehran risk category and risk score were well-balanced (right panel in Fig 1A and B). Our primary analysis, which was based on the IPTW-adjusted cohort and accounted for the Mehran 2 risk score, demonstrated that the risk of CA-AKI was significantly lower in KPS patients than in UC patients (odds ratio = 0.19, *P* = 0.009; Figure 2).

8. Predicted and actual risks for CA-AKI
The authors should also compare the predicted and actual risks for CA-AKI in both patient groups (Supplementary Figure 2 shows only the data for the entire cohort).

Reply：

Thank you for this suggestion. We have provided the results for both patient groups in the revised S2 Fig.

**S2 Fig.** Predicted and actual CA-AKI risks in different Mehran 2 risk categories in the entire cohort (a), KPS group (b), and UC group (c).

9. The CA-AKI risk calculation
a. The authors should compare the risk of CA-AKI between groups in the unadjusted cohort (and not just in the IPTW-adjusted cohort).

Reply：

Thank you for this suggestion. The comparison of the risk of CA-AKI between the groups in the unadjusted cohort is presented in Table 2, and its odds ratio is shown in S1 Table.

Table 2 Characteristics of KPS and CA-AKI in the patients

| Variable | KPS (*n* = 19) | UC (*n* = 51) | *P* value |
| --- | --- | --- | --- |
| CA-AKI | 1 (5.3) | 10 (19.6) | 0.267 |

S1 Table Associated factors of contrast-associated acute kidney injury

| Variable | Odds ratio (95% CI) | *P* value |
| --- | --- | --- |
| Univariate analysis |  |  |
| KPS | 0.32 (0.05–2.02) | 0.225 |

b. Hydration with isotonic saline is one of the most important factors in preventing CA-AKI. This is also confirmed by the univariate analysis presented in Supplementary Table 1. Since less than 50% of patients in the standard treatment group were hydrated (compared to almost 90% in the KPS group), hydration status with isotonic saline should be included in all logistic regression analyzes (univariate and multivariate). A lower incidence of saline hydration might be associated with a higher incidence of CA-AKI in standard care patients.

Reply：

Thanks for your query. Analysis of the association between isotonic saline hydration and the risk of CA-AKI was conducted using the entire cohort, which included both the KPS and UC groups. The results indicated a protective effect (odds ratio = 0.41, *P* = 0.182), although it was not statistically significant, primarily because of the limited sample size of the study (S1 Table). In accordance with your suggestion, we have included a multivariable analysis that incorporates isotonic saline hydration (see the table below). However, these results did not show a significant protective effect against isotonic saline hydration.

| Multivariable analysis* |  |  |
| --- | --- | --- |
| Femoral access | 4.49 (1.04–19.32) | 0.044 |
| LVEF <40% | 2.43 (0.56–10.51) | 0.236 |
| Multivessel disease | 6.99 (0.38–129.55) | 0.192 |
| Isotonic saline hydration | 0.61 (0.15–2.61) | 0.509 |

c. Furthermore, if <50% of patients with moderate-to-advanced CKD receive hydration with isotonic saline, this cannot be considered the standard of care for the prevention of CA-AKI in high-risk patients.

Reply：

Thank you for your comments. The strategy for CA-AKI prevention in the UC group was not standardized in all patients, which reflects the real-world situation in the daily catheterization room. For example, the rate of hydration was significantly lower in the UC group, wherein two-thirds of patients had a history of HF. Most non-hydrated patients may be concerned about fluid overload, which reflects the real-world situation in clinical practice. Therefore, we used the term UC instead of the standard-of-care in our study. In our opinion, the non-adherence to the recommendation of guidelines or expert consensus may be caused by the experience of the physician, the complexity of the patient’s condition, and the worrisome uncertainty. Therefore, we need data from a phase IV study, which may be different from clinical trials.

10. Patient outcomes
The authors should present and compare the final outcomes in both groups of patients, not just short-term data on the incidence and risk of CA-AKI based on serum creatinine in the first 3 days after cardiac catheterization. I would be interested in data on differences in morbidity and mortality in both groups of patients, including data on the evolution of kidney function at last follow-up, progression of CKD and patient survival.

Reply：

Thank you for your comments. The definition of CA-AKI in our study was identical to that in Mehran’s study, which makes a comparable incidence rate when using the Mehran 2 risk scoring system to assess the risk of CA-AKI. (as shown in S2 Fig) This short-term CA-AKI risk was strongly associated with subsequent death in the Mehran’s report. Owing to the retrospective nature and small sample size of our study, many patients (>20%) were lost to follow-up after 3 months. Moreover, we reported long-term outcomes beyond the scope of our current study. A larger prospective study should be conducted in the near future.

Other comments

1. Authors should refrain from using non-standardized definitions or terms for kidney disease/injury. The term kidney insufficiency (KI) should be replaced by chronic kidney disease (CKD) or acute kidney injury (AKI).

Reply：

Thank you for your insightful comment. Because half of the patients in our study did not have a baseline eGFR, the proportion of either CKD or AKI was uncountable. A specific term for CKD or AKI may lead to a misunderstanding of the results of our study. However, reporting kidney function as eGFR at catheterization may be more practical in specific regions, especially in referral centers for ACS and congestive heart failure, where the baseline eGFR is usually unknown. Therefore, we believe that KI may be more suitable to represent kidney function in our patient.

We sincerely appreciate the time and effort you have taken to review our manuscript. Your insightful comments and suggestions have been invaluable in improving the quality of our work, and we are grateful for your constructive feedback. Thank you for your thoughtful contributions to this process.
