# Supplementary material for: Kidney protection strategy lowers the risk of contrast-associated acute kidney injury
Source: PLoS One. 2024 Oct 24;19(10):e0312618. doi: 10.1371/journal.pone.0312618 (PMC11500849; doi:10.1371/journal.pone.0312618)
Supplement: Attachment — Submitted filename: Response to reviewer.docx [file pone.0312618.s005.docx]

Reviewer comments:
Reviewer #1:

I still have two minor issues:
1- please refer to the only iso-osmolar contrast agent as iodixanol instead of its commercial name;

***Reply:***

Thank you for your suggestion. The Methods section has been rephrased as:

[Page 5, line 76–78] "At our institute, iodixanol, a nonionic, iso-osmolar contrast medium, was administered to patients with KI during CAG. The inclusion criteria were iodixanol administration, an eGFR of <45 mL/min/1.73 m^2^ during CAG,"

2- it is not clear if the highest sCr value along the first 72 hours has been considered to adjudicate CI-AKI, or if sCr has been evaluated on days 1 and 3 (line 125 page 8); in this last case it should be considered a further limitation.

**Reply:**

Thank you for your comment. All serum creatinine follow-ups on days 1 and 3 were conducted within the monitoring period of the first 72 hours after contrast exposure. The highest post-CAG creatinine level was recorded to determine the CA-AKI incidence. However, fluctuations in serum creatinine levels during this period may occur, and some data might go undetected, particularly in situations without frequent monitoring. This limitation could lead to an underestimation of the incidence of CA-AKI. The limitation section has been revised as follows:

[Page 20, line 277–280] "Fourth, fluctuations in post-CAG serum creatinine levels within the 72-hour monitoring period may occur, and some data might go undetected, particularly in situations without frequent monitoring. This may lead to an underestimation of the incidence of CA-AKI.".
